# Mismatch negativity in common marmosets: Whole-cortical recordings with multi-channel electrocorticograms

**DOI:** 10.1038/srep15006

**Published:** 2015-10-12

**Authors:** Misako Komatsu, Kana Takaura, Naotaka Fujii

**Affiliations:** 1 Laboratory for Adaptive Intelligence, RIKEN Brain Science Institute, 2-1 Hirosawa, Wako, Saitama 351-0198, Japan

## Abstract

Mismatch negativity (MMN) is a component of event-related potentials (ERPs) evoked by violations of regularity in sensory stimulus-series in humans. Recently, the MMN has received attention as a clinical and translatable biomarker of psychiatric disorders such as schizophrenia, and for the development animal models of these psychiatric disorders. In this study, we investigated the generation of MMN in common marmosets, which are an important non-human primate model with genetic manipulability. We recorded the electrocorticograms (ECoGs) from two common marmosets with epidurally implanted electrodes covering a wide range of cortical regions. ECoG recordings were conducted in a passive listening condition with a roving oddball paradigm. We compared the ERPs evoked by repeatedly presented standard stimuli and those evoked by the deviant stimuli. Significant differences in the ERPs were observed in several cortical areas. In particular, deviant stimuli elicited larger negative activity than standard stimuli in the temporal area. In addition, the latency and polarity of the activity were comparable to human MMNs. This is thus the first report of MMN-like activity in common marmosets. Our findings have the potential to advance future gene-manipulation studies that aim to establish non-human primate models of schizophrenia.

Mismatch negativity (MMN) is a component of event-related potentials (ERPs) evoked by violations of regularity in sensory stimulus-series in humans^1^. MMN is usually associated with auditory stimuli and elicited when a sequence of repeated stimuli (standards) is interrupted by sounds that deviate in their sensory characteristics, such as intensity, duration, or frequency (deviants). Recently, MMN has received attention from a more clinical standpoint; multiple investigators have reported MMN abnormalities in patients with psychiatric disorders such as schizophrenia^2^,^3^. Higuchi, *et al.*^4^ also reported that “at-risk mental state” (ARMS) subjects who later developed schizophrenia elicited smaller MMN amplitudes compared to those of healthy controls, while no such difference was found for ARMS subjects who did not transition to schizophrenia. These results suggest that MMN could be used as a physiological biomarker for the early diagnosis of schizophrenia.

MMN could also be a translatable biomarker that can be observed in humans and other animals using the same protocol. Indeed, MMN-like activity is observable in several animal species including rodents^5^,^6^, cats^7^,^8^, and non-human primates^9^,^10^,^11^,^12^. Accordingly, researchers have started to develop animal models of MMN in order to establish animal models of psychiatric disorders, and to investigate underlying neurobiological mechanisms. The common marmoset is one of the most promising candidates for such an animal model, due to the histological similarities between the marmoset and human brain^13^ and their genetic manipulability^14^. However, MMN in common marmosets has not yet been reported.

In this study, we investigated the generation of MMN in common marmosets. We recorded the electrocorticograms (ECoGs) from two common marmosets with epidurally implanted electrodes covering a wide range of cortical regions. ECoG recordings were conducted in passive listening condition with a roving oddball paradigm (Fig. 1b)^15^,^16^. In this paradigm, a repetitive tone-train of one frequency was followed by a train of a different frequency. The first tone of a train is considered as a deviant, which eventually became a standard stimulus after a few repetitions. The roving oddball paradigm allows us exactly the same physical properties for deviant and standard tones. We hypothesised that common marmosets would also be sensitive to this paradigm.

## Results

**Figure 1:**
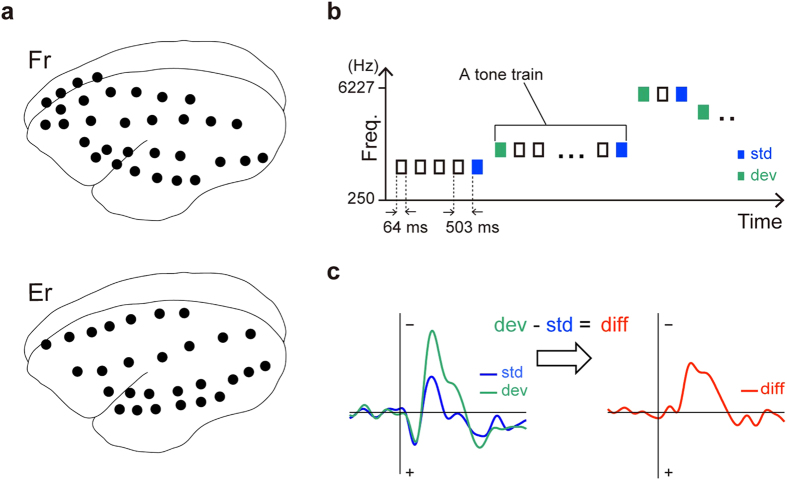
Experimental design. (**a**) Locations of the 32 electrodes in the monkey Fr and the 28 electrodes in the monkey Er were identified by computed tomography. A reference electrode was positioned on a superior part of the occipital area in both monkeys. (**b**) ECoG recordings were taken during the passive listening condition while monkeys were awake. In a roving oddball sequence, 3–11 repetitive tone-trains with 20 different frequencies were pseudo-randomly presented. We considered the last tone of a train as a standard (blue squares), and the first tone of a train as a deviant (green squares). (**c**) The difference wave was obtained by subtracting the standard ERP from the deviant ERP.

We recorded ECoGs from two common marmosets, identified as Fr and Er, with 28 and 32 epidural electrodes, respectively (Fig. 1a). In the recording sessions, 3–11 repetitive tone-trains of 20 different frequencies were pseudo-randomly presented (Fig. 1b). Because tone frequencies remained the same within each tone-train but differed between tone-trains, we considered the last tone of a train as a standard, and the first tone of a train as a deviant. Standard and deviant ERPs were calculated for each channel (Fig. 1c, left). A difference wave was obtained by subtracting the standard ERP from the deviant ERP (Fig. 1c, right).

### MMN in marmosets

**Figure 2:**
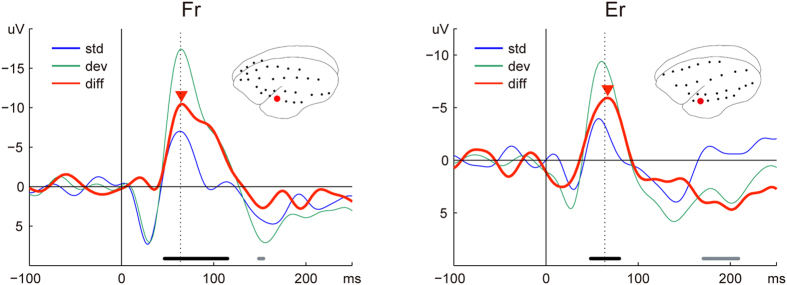
MMN in marmosets. In both monkeys, the most significant mismatch component was observed in the temporal area. The averaged waveforms of standard (blue) and deviant (green) conditions are shown, as well as the difference wave (red). The filled reverse triangles point at a negative peak of the difference wave. The horizontal bars indicate the time points, for which there is a statistically significant difference between the standard and deviant ERPs (Wilcoxon rank sum test; FDR-adjusted p-value < 0.05). The colour of the bars correspond to a negative (black) or positive (grey) polarity of the peak in the difference wave. The vertical dotted lines indicate the offset of the stimuli (64 ms after the stimuli onset). The red circle in each brain icon shows the location of the electrode from which data were obtained. A sketch of the anatomical organization of the auditory area in marmosets and the location of the electrodes relative to these areas are shown in Figure S2.

First, we calculated ERPs for standard and deviant stimuli for each channel, averaged over all trials. In order to evaluate the differences between these ERPs, we then conducted Wilcoxon rank sum tests for each channel and each time point in the range of 0–250 ms after stimulus onset. All p-values from each monkey were adjusted with the false discovery rate (FDR)^17^. For both monkeys, the most significant difference was observed in a temporal electrode (FDR-adjusted p-value = 4.6e-19 at 65 ms after onset of stimuli in Fr; FDR-adjusted p-value = 6.9e-5 at 61 ms in Er). The insets of Fig. 2 show the locations of these electrodes and waveforms in Fr (left panel) and Er (right panel), respectively. Both the standard (blue lines) and deviant (green lines) ERPs showed two positive peaks (28 ms and 164 ms for standard, and 30 ms and 156 ms for deviant tones in Fr; 30 ms and 141 ms for standard, and 27 ms and 139 ms for deviant tones in Er), as well as a negative peak (63 ms for standard, and 65 ms for deviant tones in Fr; 57 ms for standard, and 60 ms for deviant tones in Er). In both monkeys, the difference waves showed a similar negative component, which was significantly different between standard and deviant ERPs (Fig. 2, red lines). The components in Fr and Er had a time window of 40–131 ms and 37–93 ms after stimulus onset, and the latency of the negative peak was 65 and 67 ms, respectively. These profiles are comparable to MMN in humans and other species. Thus, hereafter we refer to these negative components as MMN-like activity.

### Spatiotemporal distribution of the difference between standard and deviant ERPs

**Figure 3:**
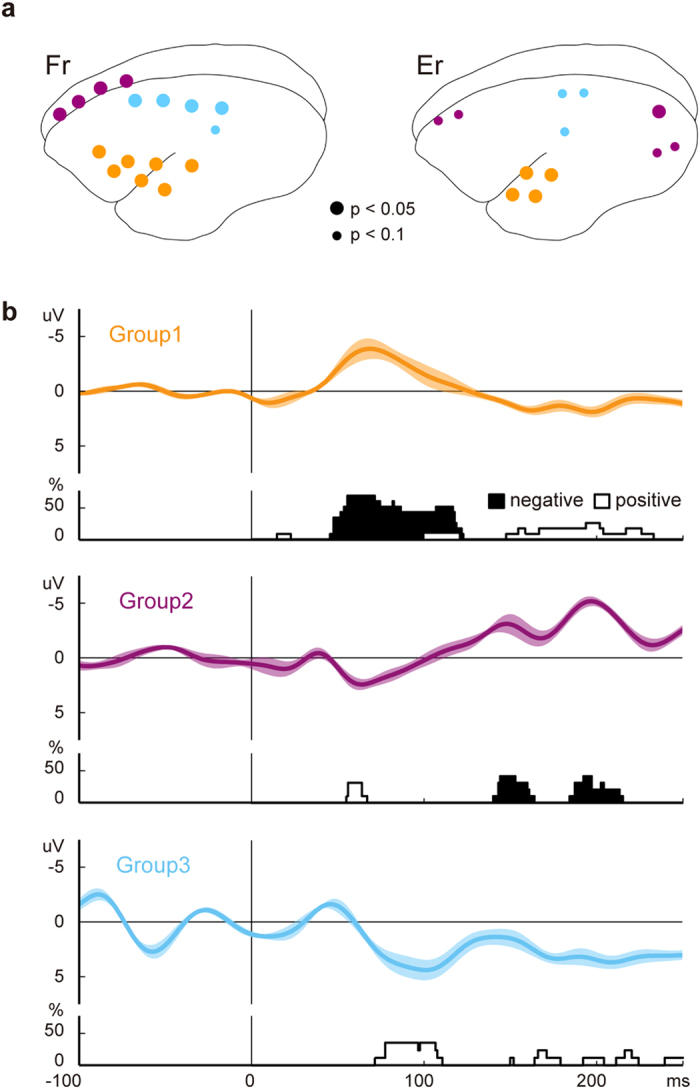
Spatiotemporal distribution of the difference between standard and deviant ERPs. (**a**) The spatial distribution of the electrodes that revealed significant differences between standard and deviant ERPs. We divided these electrodes into three groups (orange, purple, and light blue). (**b**) The grand average waveforms and the histograms of the temporal distribution of significant differences in each group. The shaded area indicates the range of SDs of the waves. The height of histograms shows the percentages of significant ERPs (FDR-adjusted p-value < 0.1) in each group at each time point. The filled and open histograms indicate the electrodes that had negative and positive components in their difference waves, respectively. The difference first occurred in temporal electrodes, and then appeared in other cortical areas.

The electrodes covered a wide range of cortex. This enabled us to see the spatiotemporal distribution of the difference between standard and deviant ERPs, unlike previous studies that have used sparsely distributed epidural electrodes^10^ or have focused on a specific cortical region with depth electrodes^9^,^11^. For monkey Fr, the standard and deviant ERPs recorded at the temporal, frontal, and parietal electrodes showed significant differences (FDR-adjusted p-value < 0.05). We also observed significant ERP differences at the temporal and occipital electrodes in monkey Er. Furthermore, ERPs at the frontal and parietal electrodes in Er showed a similar tendency as those electrodes in Fr, and they satisfied the statistical criterion of FDR-adjusted p-value < 0.1. The spatial distribution of these electrodes and individual difference waves are shown in Fig. 3a and S3, respectively. To characterize the spatiotemporal profile, we divided these electrodes into three groups based on similarities of their difference waveforms (see Methods).

Figure 3b shows the grand average waveforms and histograms of the number of electrodes that showed significant negative or positive differences at each time point in each group. The largest electrode group (Group1, orange dots in Fig. 3a) was composed of the electrodes around the lateral sulcus. These electrodes showed the negative difference (filled histogram) at 46–122 ms after stimulus onset. The difference waves during this period peaked at 81.3 ± 20.9 ms (mean ± SD). We thus considered these as MMN-like activity. An electrode showed an early positive difference (open histogram) at 15–22 ms and some showed late differences at 100–231 ms after stimulus onset. The second group (Group2, purple dots) consisted of the electrodes in frontal and occipital areas. After stimulus onset, this group showed two negative differences (at 140–163 ms and at 184–214 ms, respectively). A positive difference was observed at the electrodes in frontal area at 55–66 ms, but not at electrodes in occipital area. The electrodes in Group3 (light blue dots) were over the parietal area. These electrodes showed positive differences at 71–250 ms. We should note that the significant differences between standard and deviant ERPs occurred earlier in the electrodes around the lateral sulcus than they did in other cortical areas.

## Discussion

The present study provides the first demonstration of MMN-like activity in common marmosets. We observed negative components of the difference waves in temporal areas, which occurred 50–120 ms after the onset of the stimuli and had peaks at around 80 ms. The human MMN is well documented as a fronto-central negative potential with a latency of 100–200 ms after the onset of stimuli^18^. The latency in macaque monkey MMN-like activity (60–150 ms^10^,^11^, 50–120 ms^12^) is between that of the cat (40–80 ms^8^, 30–70 ms^7^) and the human MMN. Javitt, *et al.*^10^ attributed this MMN latency difference between macaque monkeys and the other species to the difference in size and complexity of the brain. According to their hypothesis, a larger brain size and/or more complex network causes a longer MMN latency. In the present study, the latency of MMN-like activity in marmosets was later than that previously reported for cats, despite the fact that their brain is smaller^19^. This suggests that latencies of MMN might depend on the complexity of the network of the brain rather than its size. If this is indeed the case, the similar MMN latencies of common marmosets and macaque monkeys suggest that common marmosets have a similar network complexity to macaque monkeys. The common marmoset could therefore be a promising non-human primate model of the MMN response.

Furthermore, the high spatial resolution of ECoG enabled us to investigate the spatiotemporal profiles of the difference waves across a wide range of cortex. The difference between standard and deviant ERPs first occurred in electrodes around the lateral sulcus, and then in other cortical areas, including frontal and parietal areas. This suggests that mismatch-related activity, which conveys information about a violation in the regularity of a repetitive sequence of tones, originated in areas around the lateral sulcus in common marmosets. This conclusion is compatible with evidence in human MMN^20^,^21^, and MMN-like activity in cats^8^ and macaque monkeys^10^,^11^,^12^. However, further studies are needed to clarify the relationship between the MMN-like activity in the temporal area and other mismatch-related activity.

Our finding of MMN-like activity in common marmosets provides a new perspective for the development of non-human primate models of MMN. In future studies that compare the marmoset to human/macaque MMN, it would be important to investigate the effects of the N-methyl-D-aspartate (NMDA) antagonist on MMN. In humans, it has been reported that NMDA antagonists, such as ketamine, elicit many symptoms of schizophrenia, including reductions in the MMN, when administered to normal subjects^22^. Moreover, Gil-da-Costa *et al.*^12^ have shown reductions in nonhuman primate (NHP) MMN using sub-anaesthetic administration of ketamine to macaque monkeys. Research on the NMDA-sensitivity of marmosets MMN would provide a comparative perspective across the non-human primate models, which would contribute to better understanding of MMN and to the development of the animal model of psychiatric disorders. The present study constitutes the prerequisite for these comparative studies.

## Methods

### Subjects

We used two adult male common marmosets (*Callithrix jacchus*) that weighed 320–380 g. Monkeys were implanted with the ECoG electrode array under general anaesthesia, and all efforts were made to minimize suffering. All surgical and experimental procedures were performed in accordance with the National Institutes of Health Guidelines for the Care and Use of Laboratory Animals and approved by the RIKEN Ethical Committee (No. H26-2-202).

### Implantation of ECoG arrays

Chronically implanted, customized multichannel ECoG electrode-arrays (Fig. S1) (Cirtech Inc., Japan)^23^ were used for neural recordings. Each electrode contact was 1 mm in diameter and had an inter-electrode distance of 2.5–5.0 mm. We implanted 32 (monkey Fr) and 28 (monkey Er) electrodes in the epidural space of the left hemisphere. The electrode-array covered the frontal, parietal, temporal, and occipital lobes. The additional 4 electrodes of Monkey Fr covered part of the right frontal lobe. The animals were initially sedated with butorphanol (0.2 mg/kg i.m.), and surgical anaesthesia was achieved with ketamine (30 mg/kg i.m.) and medetomidine (350 μg/kg i.m.). The animals were then positioned in a stereotaxic frame (Narishige, Japan) and placed on a heating pad during surgery. Vital signs were monitored throughout surgery. Implantation of the electrode-arrays involved the removal of a bone flap (~2 cm along the anterior-posterior axis and ~1 cm along the mediolateral axis) over the parietal cortex. The array was advanced onto the epidural space. After positioning the electrode-array, connectors were attached to the bone using dental acrylic and titanium screws (size 1.0 × 0.1 mm). The reference electrodes were placed in the epidural space and the ground electrodes in the episkull space. The anti-inflammatory corticosteroid dexamethasone (1.25 mg/kg, i.m.) was administered after surgery to prevent brain swelling. The animals were given antibiotics and analgesics daily for 5 days after surgery. Following the animals’ recovery, the position of each electrode in the arrays was identified based on a computer tomography, and then co-registered to a template T1-weighted anatomical magnetic resonance image (http://brainatlas.brain.riken.jp/marmoset/)^24^ using MRIcron software (http://www.mricro.com). In both monkeys, the electrode-array covered the frontal, parietal, occipital, and temporal cortices, including the primary auditory area (Fig. 1a and S2).

### Stimuli and task

We adopted a roving oddball paradigm. The trains of 3, 5, or 11 repetitive tones of 20 different frequencies (250–6727 Hz with intervals of 1/4 octave) were pseudo-randomly presented. Tones were identical within each tone-train, but differed between tone-trains. Because tone-trains followed on from one another continuously, the first tone of a train was considered to be a deviant tone, because it was of a different frequency from that of the preceding train. The final tone was considered to be a standard tone, because it was preceded by several repetitions of this same tone. To avoid analytical artefacts stemming from differences in the number of standard and deviant stimuli, we considered only the last tone of a train as standard. Standards and deviants were presented 240 times in each recording session. We used 64 ms (7 ms rise/fall) pure sinusoidal tones. Stimulus onset asynchrony was 503 ms. Stimulus presentation was controlled by MATLAB (MathWorks Inc., Natick, MA, USA) using the Psychophysics Toolbox extensions^25^,^26^. Tones were presented through two audio speakers (Fostex, Japan) with an average intensity of 60 dB SPL around the animal’s ear.

### ECoG recording and preprocessing

ECoG recordings were taken in the passive listening condition while monkeys were awake. In each recording session, the monkey was held in a drawstring pouch, which was stabilized in dark room. The monkey’s head was kept outside of the top of the pouch, while the monkey’s trunk was roughly stabilized through an animal jacket in the pouch. The length of each experiment was about 15 min: the first 3 min of data were used for many standard stimuli (data are not shown in this paper) and the remaining 12 min of data were used for the roving oddball sequences. Data from 3 sessions were used for analysis, which resulted in 720 (=240 × 3) standard and deviant presentations.

ECoG signals were recorded at a sampling rate of 1 kHz per channel. In the signal preprocessing, those signals were re-referenced using a common median reference (CMR) montage, and band-pass filtered from 0.1 to 30 Hz. We segmented datasets from −603 to 100 ms relative to the onsets of the deviants, so that each segment would include a deviant and a standard immediately preceding the deviant. In order to remove the segments containing outliers, we calculated a standard deviation (SD_i_, i = 1–720) for each segment and for all segments (SD_all_) and rejected segments with a SD_i_ above 3SD_all_. Two channels of monkey Fr had 1/720 and 73/720 rejected segments, respectively. After outlier rejection, the segments were divided into standard epochs and deviant epochs. For both the standard and deviant epochs, we applied a baseline correction by subtracting the mean of the 100 ms period before the stimulus onset. The standard and deviant ERPs were then calculated for each channel with all epochs. A difference wave was calculated by subtracting the standard ERP from the deviant ERP. Parts of the dataset are shared in the public server Neurotycho.org (http://neurotycho.org/)^27^.

### Electrode clustering

The electrodes that had exhibited statistically significant differences between standard and deviant ERPs were divided into groups based on the similarities of their difference waveforms. To extract trends of signals recorded across a wide range of cortex, we performed a hierarchical cluster analysis on the correlation coefficients between difference waves. A cluster tree of each correlation matrix was calculated using Ward’s method, which minimizes variance using the inner squared distance of clusters. The dissimilarity between electrodes i and j was denoted by 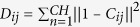. Here, CH and *C*_*ij*_ are the number of electrodes and the correlation coefficient between difference waves at electrodes i and j, respectively. By avoiding the clusters of single units and minimizing the total within-cluster variances, we obtained the cluster organizations consisting of 3 clusters for each monkey. For both monkeys, anatomically neighboring electrodes were divided into the same clusters, but excluding the electrodes in the frontal and occipital areas, of monkey Er.

## Additional Information

**How to cite this article**: Komatsu, M. *et al.* Mismatch negativity in common marmosets: Whole-cortical recordings with multi-channel electrocorticograms. *Sci. Rep.* **5**, 15006; doi: 10.1038/srep15006 (2015).

## Supplementary Material

Supplementary Figure S1

Supplementary Figure S2

Supplementary Figure S3
