# Supplementary material for: Mismatch negativity in common marmosets: Whole-cortical recordings with multi-channel electrocorticograms
Source: Sci Rep. 2015 Oct 12;5:15006. doi: 10.1038/srep15006 (PMC4601015; doi:10.1038/srep15006)
Supplement: Supplementary Figure S1 [file srep15006-s1.pdf]

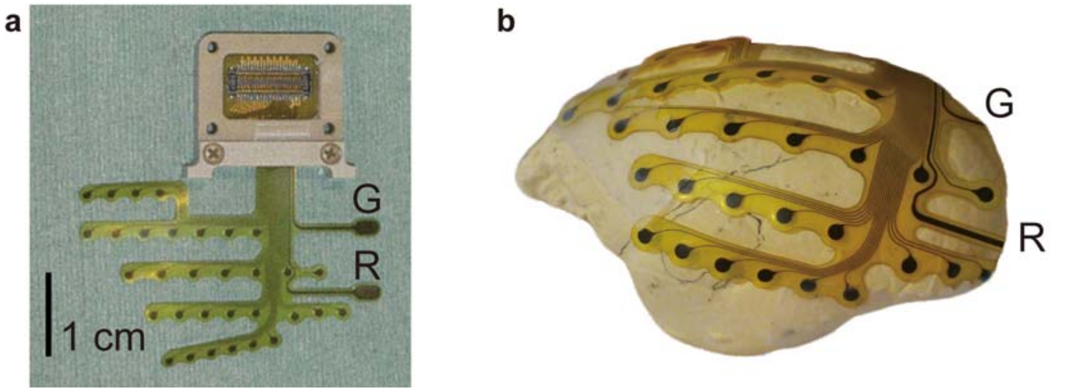

Supplementary Figure S1. The ECoG electrode array. (a) The complete electrode array and connector viewed from front. (b) A fitting example of the array on a model brain.
