# Supplementary material for: Mismatch negativity in common marmosets: Whole-cortical recordings with multi-channel electrocorticograms
Source: Sci Rep. 2015 Oct 12;5:15006. doi: 10.1038/srep15006 (PMC4601015; doi:10.1038/srep15006)
Supplement: Supplementary Figure S2 [file srep15006-s2.pdf]

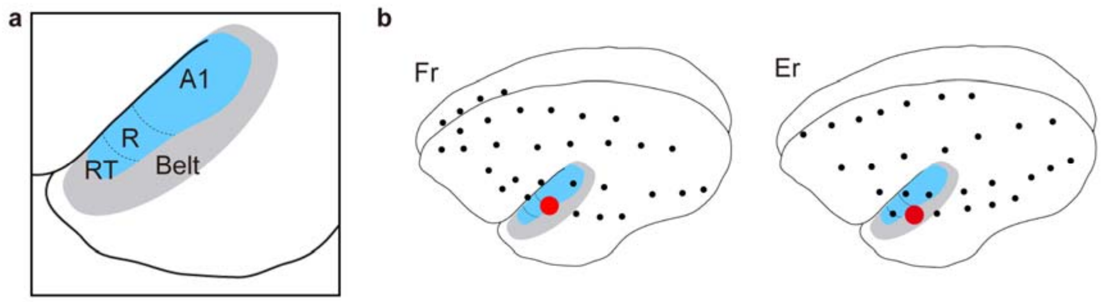

Supplementary Figure S2. Anatomical organization of auditory cortex and the location of the electrode arrays. (a) An enlarged view of the temporal area of marmoset, showing the core (A1, R, and RT) and belt areas. The borders between each auditory area are estimated by overlaying the Common Marmoset Brain Atlas (http://brainatlas.brain.riken.jp/marmoset/modules/xoonips/listitem.php?index_id=66) on standard brain. (b) Locations of the 32 electrodes in the monkey Fr and the 28 electrodes in the monkey Er. The red circles indicate the most significant electrodes in each monkey.

Legend: A1, Primary auditory cortex; R, Area R (rostral auditory cortex; RT, Area RT (rostrotemporal auditory cortex).
