# Supplementary material for: Mismatch negativity in common marmosets: Whole-cortical recordings with multi-channel electrocorticograms
Source: Sci Rep. 2015 Oct 12;5:15006. doi: 10.1038/srep15006 (PMC4601015; doi:10.1038/srep15006)
Supplement: Supplementary Figure S3 [file srep15006-s3.pdf]

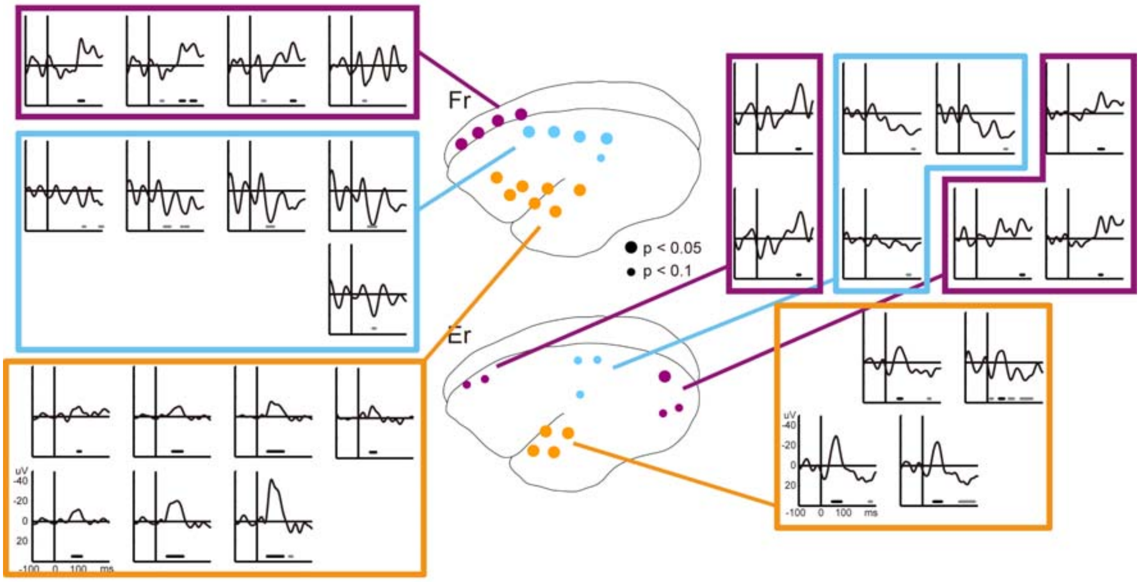

Supplementary Figure S3. Individual difference waveforms. The individual difference waveforms at the electrodes that revealed significant difference between standard and deviant ERPs are shown. The vertical bars indicate the onset of the stimuli.
